# Functional Genomics of *Aspergillus oryzae*: Strategies and Progress

**DOI:** 10.3390/microorganisms7040103

**Published:** 2019-04-10

**Authors:** Bin He, Yayi Tu, Chunmiao Jiang, Zhe Zhang, Yongkai Li, Bin Zeng

**Affiliations:** Jiangxi Key Laboratory of Bioprocess Engineering and Co-Innovation Center for In-vitro Diagnostic Reagents and Devices of Jiangxi Province, College of Life Sciences, Jiangxi Science & Technology Normal University, Nanchang 330013, China; hebin.li@foxmail.com (B.H.); tuyayi@126.com (Y.T.); jiangcm0810@jxstnu.edu.cn (C.J.); zz2007138221@163.com (Z.Z.); lyk2018@jxstnu.edu.cn (Y.L.)

**Keywords:** *Aspergillus oryzae*, functional genomics, selection markers, transformation strategies

## Abstract

*Aspergillus oryzae* has been used for the production of traditional fermentation and has promising potential to produce primary and secondary metabolites. Due to the tough cell walls and high drug resistance of *A. oryzae*, functional genomic characterization studies are relatively limited. The exploitation of selection markers and genetic transformation methods are critical for improving *A. oryzae* fermentative strains. In this review, we describe the genome sequencing of various *A. oryzae* strains. Recently developed selection markers and transformation strategies are also described in detail, and the advantages and disadvantages of transformation methods are presented. Lastly, we introduce the recent progress on highlighted topics in *A. oryzae* functional genomics including conidiation, protein secretion and expression, and secondary metabolites, which will be beneficial for improving the application of *A. oryzae* to industrial production.

## 1. Introduction

*Aspergillus oryzae* (*A. oryzae*) has been used for the production of traditional fermentation such as soy sauce, miso, and douchi for more than ten centuries. The fermentation and post-processing technologies for *A. oryzae* have matured. Additionally, *A. oryzae* has been listed as “Generally Recognized as Safe (GRAS)” by the Food and Drug Administration (FDA), and its safety has been supported by the World Health Organization (WHO) [1]. Due to its safety, *A. oryzae* is also used for the production of primary and secondary metabolites. Moreover, its strong capacity for the production of various hydrolytic enzymes makes it an ideal model for the research of gene expression and protein secretion [2]. However, the progress in *A. oryzae* functional genomics remains relatively limited. The main reasons are that few selection markers are available for *A. oryzae*, and it is not amenable to classical genetic manipulation procedures [3]. Therefore, the exploitation of effective selection markers and genetic transformation methods are of great significance. In this review, we describe the recent progress in selection markers and genetic transformation methods used for *A. oryzae*. The advantages and disadvantages of different transformation methods are also described in detail. Furthermore, the progress of highlighted topics in *A. oryzae* functional genomics such as conidiation, protein secretion and expression, and secondary metabolites are here reviewed.

## 2. Genome Sequencing of *A. oryzae*

With the development of next-generation sequencing technology, genome sequencing has become increasingly common—especially for fungi. Due to its small genome size and ease of sequencing and annotation, *A. oryzae* has been recognized as a model filamentous fungus of genomic research, which provides theoretical foundation for the research of functional genomics, expression regulation mechanism and evolution [4]. In 2005, sequencing of the *A. oryzae* RIB40 genome was accomplished by Japanese scientists through the whole-genome shotgun (WGS) approach [5]. The 37 Mb genome was predicted to contain eight chromosomes, which were comprised of 12,074 genes encoding proteins with a length greater than 100 amino acid residues. Comparing to *Aspergillus fumigatus* and *Aspergillus nidulans*, *A. oryzae* has about 34% and 29% larger genome sizes, respectively. The unique genes mainly refer to secretory hydrolases, stress responses, as well as primary and secondary metabolism [6]. Since the release of the *A. oryzae* RIB40 genome sequence, more efforts have been involved in *A. oryzae* genome projects. Furthermore, third-generation sequencing technology has accelerated the progress of *A. oryzae* genome projects. This sequencing technology is characteristic of long reads, which can reduce the errors of genome assembly and improve the quality of genome annotation. To date, approximately ten *A. oryzae* strains have been sequenced and deposited in the public NCBI database. The genome sizes of these strains range from 35.42 to 41.16 Mb, and the GC content of most strains is approximately 48% (Table 1). Although the genomes of so many *A. oryzae* strains have been decoded, the functional genomics is still in its infancy for the breeding of industrial strains. Among the more than 10,000 genes, only around 200 genes are functionally verified, accounting for 1.7% of the whole genome.

## 3. Strategies for Functional Genomics of *A. oryzae*

### 3.1. Selection Markers

Effective selection markers are the basis of functional genomics research, which can reduce false positive rates and reduce the workload for screening. Drug resistance markers and auxotrophic markers are the two main selection markers used for transformation vectors in *A. oryzae*.

#### 3.1.1. Drug Resistance Markers

Drug resistance markers are the most widely used markers for fungal genetic transformation, which do not require the host strain to be auxotrophic, and can be used as dominant selection markers. After transforming the vector with drug resistance genes, the host strain can grow in the presence of specific drug concentrations. However, there are very few foreign hetero gene markers that can be used as transformation markers in *A. oryzae* since it has high native drug resistance [3]. Hygromycin B resistance genes such as *hph* and *hygr* are the most widely used drug resistance markers in *A. oryzae* genetic transformation. For example, Fernandez et al. successfully constructed an interference vector for *A. oryzae* and repressed the expression of the *wA* gene involved in the color of spores using the hygromycin B resistance gene *hph* as a selection marker [13]. Besides, the bleomycin (Bm) resistance marker has also been used in the transformation of *A. oryzae*. Satoshi at al. established the transformation system by Bm selection for *A. oryzae* by increasing the susceptibility of *A. oryzae* to Bm [14]. Furthermore, pyrithiamine (PT) and phleomycin resistance genes have also been applied as dominant selection markers for the transformation of *A. oryzae* [15,16]. However, most transformation systems with drug resistance markers require expensive antibiotics. Meanwhile, some resistance genes have the risk of being transferred to the environment and other microorganisms. Therefore, the FDA has banned the use of drug resistance markers in food microorganisms, including *A. oryzae* [17].

#### 3.1.2. Auxotrophic Markers

Auxotrophs show the same phenotype with the wild type after transforming the vector with the corresponding auxotrophic marker. pyrG is the most widely used drug resistance marker used for *A. oryzae* genetic transformation. In filamentous fungi, the *pyrG* gene encodes for orotidine-5’-monophosphate (OMP) decarboxylase, which is a key enzyme for the biosynthesis of uridine/uracil (necessary for fungal survival). Therefore, the pyrG mutants are unable to convert orotidine into uridine and require additional supplementation of uridine or uracil for their growth [18]. Yasuda et al. established an efficient gene knockout system using *A. oryzae* KBN630 as an original strain and *pyrG* as a selection marker [19]. Nguyen et al. confirmed that the *pyrG* selectable marker is a powerful tool for genetic transformation and recombinant gene expression studies in *A. oryzae* [20]. Maruyama et al. and Yoon et al. knocked out multiple genes through a *pyrG* selectable marker [21,22]. Other nutritional markers were also exploited for transformation systems of *A. oryzae*, such as *niaD* for nitrate assimilation, *adeA* for adenine, or *argB* for arginine biosynthesis, etc. (Table 2). The screening of transformants with auxotrophic markers is convenient and efficient, based on selective culture media. In summary, transformation systems for *A. oryzae* have mainly been developed based on auxotrophic markers for the complementation of auxotrophic mutant strains.

### 3.2. Strategies for the Transformation of A. oryzae

Efficient strategies for the transformation of *A. oryzae* are an important foundation for its functional genomic investigation. However, the transformation efficiency of *A. oryzae* is low when compared to *Saccharomyces cerevisiae* (*S. cerevisiae*). The cell wall of *A. oryzae* is the main obstacle to successful genetic transformation [32]. Various transformation methods for *A. oryzae* have been established, including protoplast-mediated transformation (PMT), *Agrobacterium*-mediated transformation (AMT), and electroporation (EP) (Table 3).

#### 3.2.1. Protoplast-Mediated Transformation

**Figure 1:**
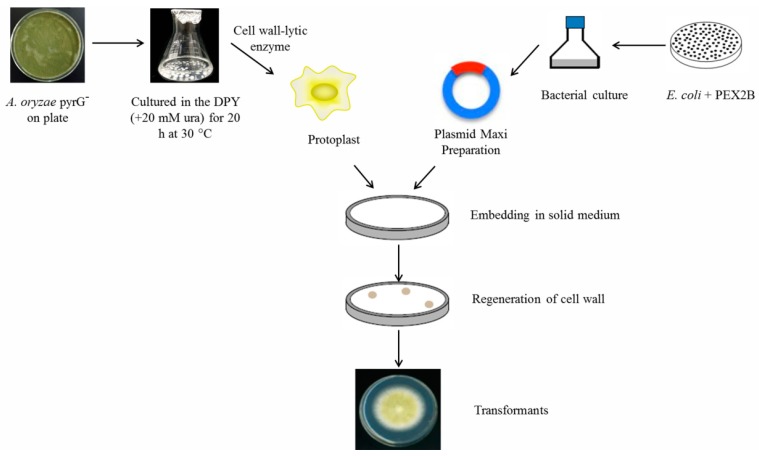
Transformation process of *A. oryzae* using protoplasts. All procedures are performed in the presence of an osmotic stabilizer. DPY: dextrin–peptone–yeast medium. ura: uracil.

The main strategy for introducing DNA into fungi is the PMT method, which has been successfully applied to a large number of fungal species [33]. This method has been successfully applied for the transformation of *A. oryzae* due to the ease of obtaining homozygotes. For example, Murakami et al. introduced an aspartic proteinase from *Mucor pusillus* into *A. oryzae* by PMT using the *niaD* gene as the selective marker [34]. In addition, Tada et al. transformed a neutral ceramidase orthologue into *A. oryzae* using the PMT method [35]. The procedure for *A. oryzae* is a modified version according to the method of other fungi [36]: (1) The pyrG^−^ strain is inoculated in 100 mL dextrin–peptone–yeast (DPY) liquid medium (2% dextrin, 1% polypeptone, 0.5% yeast extract, 0.5% KH_2_PO_4_, 0.05% MgSO_4_·7H_2_O, pH 5.5) containing 20 mM uridine and 0.2% uracil. (2) Protoplast formation is performed through incubation of the mycelia in 10 mL transformation (TF) Solution I (50 mM maleic acid, 1% Yatalase, 0.6 M (NH_4_)_2_SO_4_.) containing cell wall-lytic enzyme and checked by microscopic observation. (3) The DNA fragment containing the target gene is mixed with the protoplast suspension under PEG-CaCl_2_ conditions. (4) Transformants are visible on the agar medium after 3–4 days cultivation at 30 °C. They are inoculated onto new minimum+methionine (M+Met) agar medium (0.2% NH_4_Cl, 0.1% (NH_4_)_2_SO_4_, 0.05% KCl, 0.05% NaCl, 0.1% KH_2_PO_4_, 0.05% MgSO_4_⋅7H_2_O, 0.002% FeSO_4_⋅7H_2_O, 2% glucose, 0.15% methionine, pH 5.5) to obtain single colonies or axenic cultures (Figure 1). In this procedure, the key step is protoplast preparation, which requires removal of the cell wall. At present, cell wall-lytic enzymes are commonly used, while some other methods such as mechanical and other non-enzymatic methods have also been reported [37]. Therefore, the transformation rate is mainly affected by the efficiency of different batches of cell wall-degrading enzymes in filamentous fungi [38]. Furthermore, due to the varying cell wall composition and possible defense mechanisms, PMT protocols cannot be generalized across various fungi [39].

#### 3.2.2. *Agrobacterium*-Mediated Transformation

**Figure 2:**
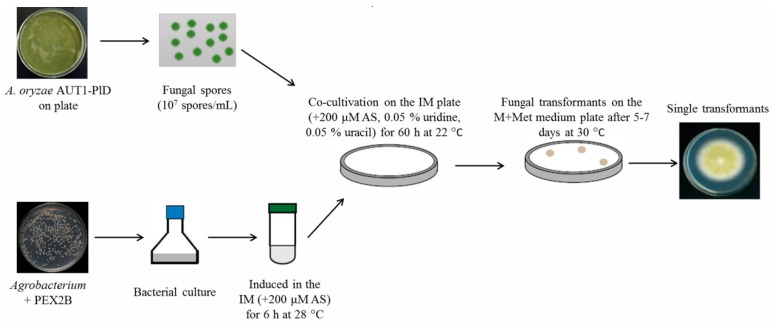
Transformation of auxotrophic *A. oryzae* using the AMT method. IM: induction medium.

Another strategy for gene targeting in *A. oryzae* is AMT, which is a fundamentally different method to PMT that is used for transformation. This method was traditionally used in plants and was later applied to yeast and filamentous fungi [40,41,42]. *Agrobacterium tumefaciens* is a Gram-negative bacterium that can transfer the T-DNA region of the Ti plasmid to the genome of the infected plant. The T-DNA region is bordered by two imperfect inverted repeats which interact with a set of virulence proteins (located in the Ti plasmid) that facilitate the DNA transference to the host. The T-DNA and the selection marker are integrated randomly in the genome of transformants [43]. Fungal spores, germlings, and mycelia have been reported to be successfully transformed using this method [44,45]. The first step of the AMT procedure for *A. oryzae* is the introduction of the PEX2B vector into *A. tumefaciens* cells. Then, a mixture of the spore suspension and the induced *A. tumefaciens* suspension are spread on the induction medium (IM) agar plate containing 200 μM AS, 0.05% uridine, and 0.05% uracil. Lastly, the membrane is transferred to the M+Met medium plate (Figure 2). Nguyen et al. constructed versatile binary vectors carrying *pyrG* auxotrophic marker and fluorescent reporter genes and transformed them into *A. oryzae* RIB40 using the AMT method [24]. In 2017, Nguyen et al. further confirmed that the use of AMT with the *pyrG* selectable marker is a powerful and efficient method for genetic transformation and recombinant gene expression studies in *A. oryzae* [20]. According to the AMT method introduced by Nguyen et al., our group established a dual selection marker transformation system using AMT for *A. oryzae* 3042 [23]. The AMT method is more efficient and simpler to implement due to the direct use of fungal spores as the material, avoiding the need to obtain protoplasts, which is laborious and requires a tricky procedure. Furthermore, the AMT method can improve the efficiency of gene deletion in some fungi in comparison with other transformation methods, and improves targeted integration. However, there are also some disadvantages: it is difficult to develop adequate binary vectors containing the *vir* genes and the heterologous DNA, and AMT protocols cannot be generalized across various fungi due to the varying parameters during *Agrobacterium*–fungi conjugation, which affects the transformation rate.

#### 3.2.3. Electroporation

Electroporation is a method that applies a high-voltage electric pulse to a solution containing protoplasts and DNA. The electroporation of protoplasts has been achieved for several fungi, such as yeast and *Aspergillus unguis* [46,47]. However, the electroporation protocols need to be optimized across various fungi and are relatively complicated. Furthermore, although relatively fast and simple, electroporation provides a low DNA transfer efficiency (∼1–5 × 10^4^ colonies/μg) in comparison with PMT (1 transformant per 10^5^ spores) and AMT (10 transformants per 10^5^ spores) methods [48]. Researchers have attempted to transform *A. oryzae* through electroporation, but failed. Therefore, there are no reports on the implication of electroporation methods for *A. oryzae* transformation.

### 3.3. Genetic Manipulations

To determine gene functions or enhance the ability of a strain to produce a certain component (e.g., protein production), it is important to generate a host with effective selection markers and high transformation rate applicable to genetic manipulations. However, compared with other fungi (e.g., yeasts and *Aspergillus nidulans*), only a few attempts to manipulate genes had been performed in *A. oryzae*. Xu et al. breed novel food-grade industrial koji molds with high activity of acid protease by interspecific genome recombination between *A. oryzae* and *A. niger* [49]. Bocking et al. generated highly branched mutants by UV or nitrous acid mutagenesis [50]. Moreover, Yamada et al. constructed an RNAi system for gene silencing in *A. oryzae*. In this system, compatible restriction enzyme sites and the Gateway system were used to create the hairpin RNA cassette [51]. Recently, the bacterial and archaeal immune mechanism CRISPR/Cas9 was engineered into a powerful gene editing system, which contained only two components: the Cas9 nuclease and a single guide RNA (sgRNA). The CRISPR/Cas9 system has been successfully adapted to filamentous fungi such as *Aspergillus aculeatus*, *Aspergillus fumigatus*, and *A. oryzae* [52,53]. The procedure of this system is simple and efficient. Firstly, codon usage of *cas9* is optimized and then the optimized *cas9* is inserted into expression vector. Secondly, the sgRNA sequence is fused with promoter and inserted into the expression vector containing *cas9*. Katayama et al. developed the CRISPR/Cas9 system in *A. oryzae* to knock out *wA* (polyketide synthase), *pyrG*, and *yA* (p-diphenol oxidase). Because the isolation of *A. oryzae* conidia containing only mutated nuclei is difficult, the mutational rates ranged from 10% to 20%, which was lower than those of A. *fumigatus* [54]. Therefore, this system for genome editing used in *A. oryzae* was still at beginning.

## 4. Advances in the Functional Genomics of *A. oryzae*

Based on the characteristics and industrial application of *A. oryzae*, the functional genomics of *A. oryzae* is mainly focused on three areas of study: conidiation, protein secretion and expression, and secondary metabolites.

### 4.1. Conidiation

Conidiation is a common event occurs in the asexual developmental process of *Aspergillus* species. In this process, more than 10,000 conidia can be produced in multicellular organs called conidiophores [55]. The conidia of *A. oryzae* are essential for the food industry, as they are often used as starters in the first step of fermentation to digest ingredients [56]. Therefore, increasing interest has been focusing on the investigation of the conidiation regulatory pathway. Conidiation is putatively induced by external or internal signals that activate a genetic program of sporulation. Studies have shown that conidiation is induced when nutrients in culture medium are limited, such as limitation of carbon and nitrogen sources [57]. Moreover, Hatakeyama et al. reported that conidiation is repressed by white and red light [58]. The conidiation regulatory mechanism has been well-studied in *A. nidulans*. Experimental evidence has shown that the regulatory mechanisms between *A. nidulans* and *A. oryzae* are conserved. *BrlA* is the most-investigated conidiation regulatory gene, which activates a central regulatory pathway controlling the expression of conidiation-specific genes such as *abaA*, *wetA*, stuA, *medA*, and *vosA*. *BrlA* disruptants of *A. oryzae* showed the inability to form conidiophores. Moreover, the fadA G-protein-dependent signaling pathway also regulates conidiation by suppressing *brlA* activation. The Fad-mediated signaling is regulated by *FlbA*, a specific regulator of G-protein signaling. The *fadA* mutant in *A. oryzae* causes reduction of intrinsic GTPase activity and results in uncontrolled vegetative growth and autolytic phenotypes. Additionally, the overexpression of Rim15p (a serine-threonine kinase) in *A. oryzae* showed reduced conidiation, and conidiation was completely abolished in the *Rim15p* deletion strain [59].

### 4.2. Protein Secretion and Expression

Compared to other eukaryotic expression systems, such as yeast, algae, or insect cells, *A. oryzae* possesses a high capacity for product secretion. Thus, *A. oryzae* is an excellent host for industrial protein production, including glucose oxidases, amylases, chymosin, pectinases, catalases, cellulases, proteases, phytases, lipases, and xylanases in food [60]. These products synthesized by *A. oryzae* are easier to accept than those produced by non-approved production hosts, since *A. oryzae* has been listed as GRAS by FDA [1]. Therefore, *A. oryzae* has been receiving increased attention as a host for expressing homologous and heterologous proteins. However, heterologous eukaryotic proteins are generally inefficiently produced compared to endogenous proteins. To date, several difficulties have been identified and overcome in the heterologous protein production process. Firstly, the most important factors are effective selection markers and efficient strategies for the transformation of *A. oryzae*, which have been described above. Secondly, proteolytic degradation by secreted proteases into the culture medium is another significant problem in heterologous protein production [61]. To overcome this problem, researchers constructed multiple protease gene disruptants to avoid proteolytic degradation, which increased the production yields of heterologous proteins [22,62]. Furthermore, the repression of vacuolar protein sorting (*VPS*) and autophagy genes, which play important roles in the secretory pathway, led to a significant improvement of heterologous protein production by *A. oryzae* [63,64]. Moreover, the genetic fusion of a target protein with endogenous carrier proteins—which are generally secreted enzymes—has also improved the yield of heterologous proteins [65]. Heterologous fusion protein is thought to improve the yields in transcription and posttranscriptional processes. A recently developed approach was based on the fusion proteins that increased the expression of heterologous protein by modulating endoplasmic reticulum-Golgi cargo receptors [62]. Lectin-type cargo receptors might affect the secretion of carrier-fused heterologous proteins and therefore improve heterologous protein production, as glycoproteins like α-amylase are commonly used as carrier proteins.

### 4.3. Secondary Metabolites

*A. oryzae* not only possesses a prominent secretory capacity, it also produces abundant secondary metabolites. A comparison of the *A. oryzae* genome with *A. nidulans* and *A. fumigatus* revealed that the additional genes are mainly involved into transporters, secretory hydrolases, and secondary metabolism genes, among which the expansion of secondary metabolism genes is most prominent. Fungal secondary metabolites are biosynthesized by gene clusters, most of which encode typical backbone enzymes, such as polyketide synthase (PKS) and non-ribosomal peptide synthase (NRPS). The *A. oryzae* genome contains 56 gene clusters, which are enriched in regions lacking synteny with those in either *A. fumigatus* or *A. nidulans* [5]. Some of these secondary metabolites have many kinds of pharmacological activities. For example, Zhou et al. isolated nine isocoumarin derivatives from solid cultures of *A. oryzae* which showed moderate inhibitory activities against several human tumor cell lines [66]. In addition, many other secondary metabolites with pharmacological activity have also been identified from *A. oryzae*, such as aspirochlorine (antifungal activity), aspergillic acids (antifungal activity, antihypertensive), and aspergillomarasmine B (antiangiotensin) [67,68]. Furthermore, penicillin biosynthetic genes have been clustered in the *A. oryzae* genome. Marui et al. generated a strain that showed a greater than 100-fold overexpression of *VeA* utilizing a strong promoter [69]. However, some mycotoxins have also been isolated from *A. oryzae*, including kojic acid, β-nitropropionic acid (BNP), and cyclopiazonic acid (CPA) [70]. Some researchers reported that kojic acid has strong inhibitory activity against tyrosinase and antibacterial activity, while recent studies have shown that kojic acid has potential carcinogenicity [71]. The 14 genes involved in the biosynthesis of kojic acid formed a cluster in the genome from AO090113000132 to AO090113000145, including a Zn(II)_2_Cy_6_ transcription factor gene (*kojR*), an enzyme gene (*kojA*), and a transporter gene (*kojT*) [72]. BNP causes sugarcane disease, and is one of the mycotoxins reported from *A. oryzae* and *A. flavus*. However, the genetic basis for the biosynthesis of BNP is not completely understood [73]. CPA is an indol tetrameric acid that was originally isolated from *A. flavus*. It has also been isolated repeatedly from *A. oryzae*, and it is possible to remove the CPA gene cluster to avoid CPA biosynthesis in biotechnological processes [74]. Moreover, the gene cluster involved in aflatoxin biosynthesis in the *A. oryzae* genome has also been studied frequently, as *A. oryzae* was probably domesticated from *A. flavus*. The aflatoxin cluster contains about 30 genes, and the roles of these genes in aflatoxin synthesis have almost been clarified [75,76]. It has been shown that *A. oryzae* cannot produce aflatoxins due to a lack of the *aflR* gene, even under aflatoxin-conducing conditions [77,78].

## 5. Conclusions

*A. oryzae* has had widespread applications in traditional fermentation processes and has promising potential to produce primary and secondary metabolites. Recent progress in next-generation sequencing technology has boosted the research on the functional genomics of *A. oryzae*, which is helpful to the genetic improvement of *A. oryzae* fermentative strains. Two types of effective selection markers including drug resistance markers and auxotrophic markers have been exploited to screen positive strains. Due to their applicability and safety, auxotrophic markers are preferential to resistance markers. Furthermore, three transformation methods of *A. oryzae* have been established for functional genomics investigation, including protoplast-mediated transformation, *Agrobacterium*-mediated transformation, and electroporation (Table 3). We believe that the coupling of protoplast-mediated transformation with *Agrobacterium*-mediated transformation will be useful to enhance transformation efficiencies. Based on the optimized selection markers and transformation strategies, the current functional genomics of *A. oryzae* is mainly focused on conidiation, protein secretion, and expression as well as secondary metabolites, which will be beneficial for better application in industrial production.

## Figures and Tables

**Table 1:** *Aspergillus oryzae* strains with completed genome sequences.

Strains	Assembly	Level	Sequencing Strategies	Size (Mb)	GC%	Gene	Ref.
RIB40	GCA_000184455	Chromosome	Whole-genome Shotgun	37.91	48.26	12,074	[5]
3042	GCA_000269785	Contig	Solexa	36.57	48.28	11,639	[7]
AS 3.951	GCA_000278405	Contig	Illumina	36.33	48.30	/	[8]
AS 3.863	GCA_000278425	Contig	Illumina	36.40	48.30	/	[8]
RIB326	GCA_000320905	Contig	SOLiD	35.42	48.40	/	[9]
100-8	GCA_000691885	Contig	Solexa	36.76	48.30	11,188	[10]
BCC7051	GCA_002007945	Scaffold	PacBio	38.50	47.20	11,456	[11]
SRCM101975	GCA_002214955	Contig	Ion Torrent	37.12	48.30	/	[3]
SRCM101989	GCA_002214965	Contig	Ion Torrent	36.97	48.30	/	[3]
ATCC 12892	GCA_002894705	Scaffold	Illumina	41.16	47.60	/	[12]

**Table 2:** Different mutants of *Aspergillus oryzae* with auxotrophic markers.

Wild Strains	Mutant Strains	Selection Marker	Ref.
3042	pyrG auxotroph	*pyrG^−^*	[23]
RIB40	pyrG auxotroph	*pyrG^−^*	[20,24]
RIB40	niaD300	*niaD^−^*	[25]
RIB40	NSR13	*argB^−^*, *adeA^−^*	[26]
RIB40	NS4	*ligD*, *pyrG^−^*	[27]
RIB40	NSR1	*niaD^−^*, *SC^−^*, *adeA^−^*	[28]
RIB40	NSlD1	*niaD^−^*, *SC^−^*, *ligD^−^*	[22]
RIB40	NSPlD1	*niaD^−^*, *SC^−^*, *pyrG^−^*, *ligD^−^*	[21]
RIB40	NS-tApE	*niaD^−^*, *sC^−^*, *adeA^−^*, *argB^−^*	[29,30]
RIB40	NSRKu70-1-1A	*niaD^−^*, *SC^−^*, *argB^−^*, *adeA^−^*, *ku70^−^*	[31]

**Table 3:** Three common strategies for the transformation of *Aspergillus oryzae.*

Methods	PMT ^a^	AMT ^b^	EP ^c^
Principles	Use cell-wall-degrading enzymes to prepare protoplasts. Uptake the DNA by the addition of PEG and CaCl_2_.	*Agrobacterium tumefaciens* is able to transfer the T-DNA region of the Ti plasmid to the genome of *A. oryzae*.	Uptake DNA is mediated by reversible membrane permeabilization induced by the local application of electric pulses.
Advantages	1. It is easier to get homozygotes because of the large number of receptor cells. 2. Spores, germlings, and mycelium can be used.	1. Low copy number of inserted DNA. 2. Spores, germlings, and mycelium can be used. 3. Improves targeted integration.	1. Simple and cheap. 2. Spores and germlings can be used.
Disadvantages	1. The transformation rate is affected by the efficiency of different batches of cell-wall-degrading enzymes. 2. Requires a regeneration procedure.	1. It is difficult to obtain adequate binary vectors containing the vir genes and the heterologous DNA. 2. Various parameters during co-cultivation affect the transformation rate.	1. The protocols need to be optimized and are relatively complicated. 2. Protoplast formation is needed.

^a^ PMT: protoplast-mediated transformation; ^b^ AMT: *Agrobacterium*-mediated transformation; ^c^ EP: electroporation.
